# Supplementary material for: Cellular senescence dysregulates antiviral interferon responses in idiopathic pulmonary fibrosis
Source: bioRxiv. 2026 Apr 23:2026.04.20.719739. Preprint. [Version 1] doi: 10.64898/2026.04.20.719739 (PMC13131623; doi:10.64898/2026.04.20.719739)
Supplement: Supplement 1 [file media-1.pdf]

Supplementary Figure 1

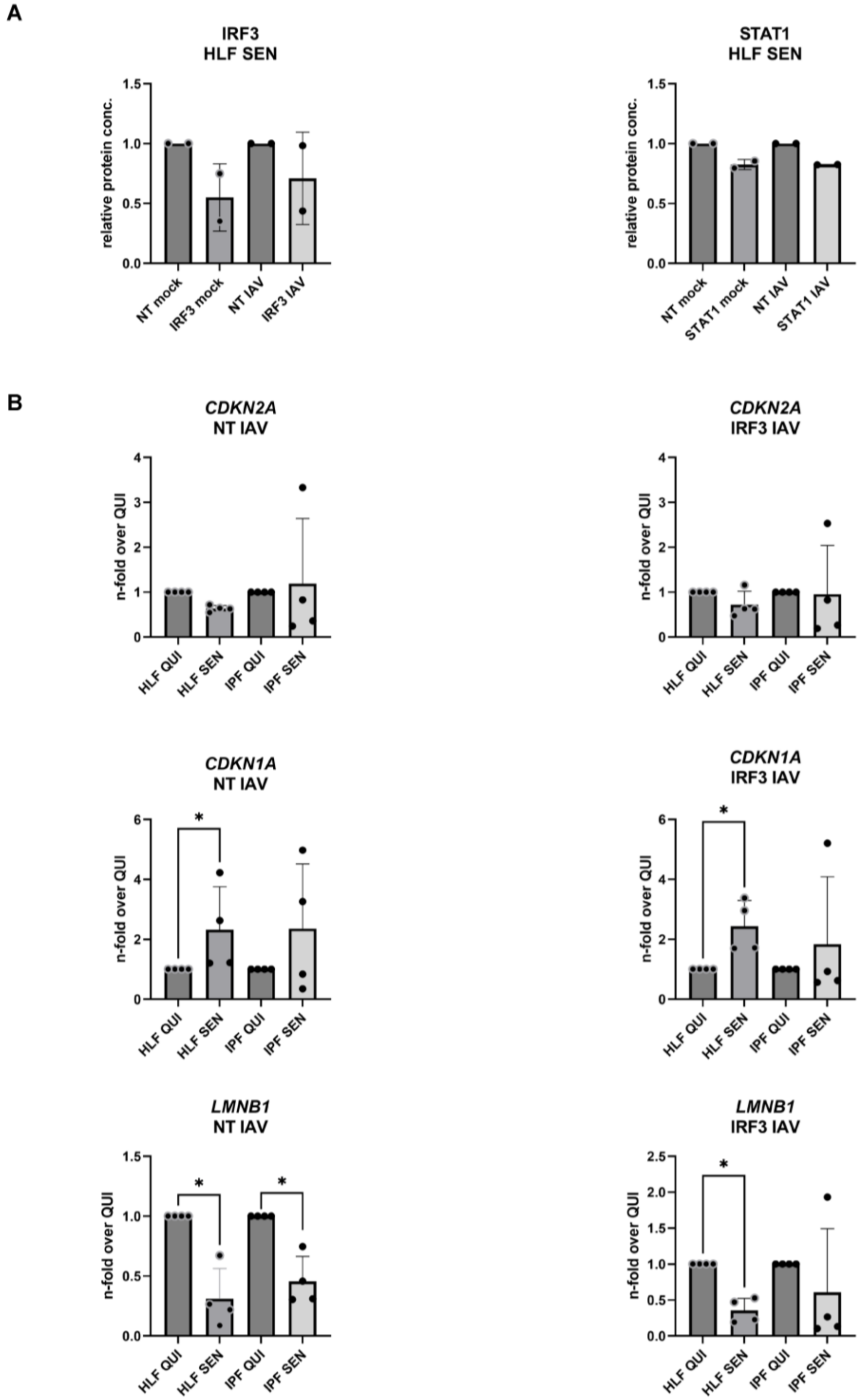

(A) Quantification of *IRF3* and *STAT1* proteins in senescent (SEN) HLF healthy lung fibroblasts via Western blot (n = 2). Protein levels were quantified using Image J and are normalized to the respective housekeeping protein (Vinculin for *IRF3* and β-Actin for *STAT1*) and are expressed relative to the respective NT control siRNA condition. (B) The relative expression of senescence markers *CDKN1A* (p21), *CDKN2A* (p16), and *LMNB1* (Lamin B1) was quantified by qRT-PCR in both infected quiescent (QUI) and SEN HLF and idiopathic pulmonary fibrosis (IPF) fibroblasts transfected with NT or *IRF3* siRNA (n = 4). Gene expression levels were normalized to β-Actin and reported relative to the respective quiescent control. Statistical significance was determined using the Mann-Whitney U test (B). * $p \leq 0.05$.

Supplementary Table 2

**Gene name**	**Forward sequence**	**Reverse sequence**
IRF3	TCTGCCCTCAACCGCAAAGAAG	TACTGCCTCCACCATTGGTGTC
STAT1	ATGGCAGTCTGGCGGCTGAATT	CCAAACCAGGCTGGCACAATTG
ACTB	CATGTACGTTGCTATCCAGGC	CTCCTTAATGTCACGCACGAT
LMNB1	TTGGATGCTCTTGGGGTTC	AAGCAGCTGGAGTGGTTGTT
CDKN1A (p21)	TCACTGTCTTGTACCCTTGTGC	GGCGTTTGGAGTGGTAGAAA
CDKN2A (p16)	CTCGTGCTGATGCTACTGAGGA	GGTCGGCGCAGTTGGGCTCC

Forward and reverse primer sequences used in qRT-PCR. All primers are human.
